# Desiccation Tolerance of *Aedes aegypti* and *Aedes albopictus* Eggs of Northeastern Argentina Origin

**DOI:** 10.3390/tropicalmed10040116

**Published:** 2025-04-21

**Authors:** Mía E. Martín, Elizabet L. Estallo, Luis G. Estrada, Carolina Matiz Enriquez, Marina Stein

**Affiliations:** 1 Centro de Investigaciones Entomológicas de Córdoba (CIEC), Facultad de Ciencias Exactas, Físicas y Naturales, Universidad Nacional de Córdoba, Córdoba 5000, Argentina; 2 Instituto de Investigaciones Biológicas y Tecnológicas (IIBYT), Consejo Nacional de Investigaciones Científicas y Técnicas (CONICET)-Universidad Nacional de Córdoba, Córdoba 5000, Argentina; 3 Instituto Nacional de Medicina Tropical (INMeT), Administración Nacional de Laboratorios e Institutos de Salud “Dr. Carlos G. Malbrán”, Ministerio de Salud de la Nación, Puerto Iguazú 3370, Argentina; 4 Instituto de Medicina Regional (IMR), Universidad Nacional del Nordeste, Resistencia 3500, Argentina

**Keywords:** vector-borne diseases, egg survival, *Aedes* mosquito, subtropical climate, Argentina

## Abstract

This study examines the desiccation tolerance of *Aedes aegypti* and *Aedes albopictus* eggs, two major arbovirus vectors, in a subtropical region of Argentina to understand their survival under varying relative humidity (RH) conditions (35%, 68%, and 82%). Laboratory experiments revealed that *Ae. aegypti* eggs exhibited significantly higher survival rates across all RH levels and exposure times compared to *Ae. albopictus*. After 1 month, *Ae. aegypti* eggs maintained 88% survival at 35% RH, while *Ae. albopictus* survival dropped to 38%. This disparity was more pronounced at low RH, where *Ae. albopictus* eggs experienced a rapid decline in survival over time. The results highlight the importance of RH as a key factor influencing the persistence of both species in the environment. The coexistence of *Ae. aegypti* and *Ae. albopictus* in Puerto Iguazú suggests that microhabitats with distinct humidity conditions may favor one species over the other. These findings provide crucial insights for predicting mosquito population dynamics under changing climate conditions and developing more effective vector control strategies to reduce arbovirus transmission in subtropical regions.

## 1. Introduction

*Aedes* (*Stegomyia*) *aegypti* (Linnaeus) and *Ae.* (*Stegomyia*) *albopictus* (Skuse) are two of the most important mosquito vectors of arboviruses globally. In Argentina, dengue has become a major public health issue, with over 529,000 cases reported during the 2023–2024 outbreak—more than triple the number from the previous year [1].

In recent decades, *Ae. aegypti* has expanded into temperate areas of Argentina, with records reaching as far south as Río Negro province (40°43′ S, 64°56′ W) [2]. Meanwhile, *Ae. albopictus*—originally from Asia—was first detected in Argentina in 1998 and remains largely restricted to northeastern provinces (27°56′ S 55°59′ W), with a limited expansion compared to other countries [3]. In the country, its expansion has not experienced the rapidity that was observed in the United States and Brazil, where in a few years, the mosquito covered thousands of kilometers from the point of its first discovery [4].

Both species adapted to urban and peridomestic environments, and lay their eggs individually on the inner surfaces of natural and artificial containers [5,6]. The eggs are highly resistant to drought, allowing them to remain viable in dry conditions for several months, and even up to a year in favorable conditions [7]. In this way, their populations endure unfavorable periods, such as cold temperatures and low rainfall, by remaining in the egg stage [8]. This ability allows species to establish themselves in a non-native habitat, facilitating long-distance transport [9].

Therefore, egg survival in unfavorable conditions is a key factor for the persistence of mosquito populations. Egg survival may be influenced by intrinsic factors such as senescence, but also meteorological conditions such as relative humidity, and the developmental stage of the embryos when exposed to dry conditions [10,11,12]. Once embryonic development is complete, hatching occurs upon water immersion. Comparative egg survival studies on *Aedes* mosquito species conducted under different RH in Asia revealed interspecific variations. *Aedes aegypti* eggs demonstrated greater resilience to prolonged desiccation across a broader humidity range, withstanding conditions as low as 42% RH [8,13]. Likewise, Florida strains of *Ae. aegypti* exhibited low egg mortality regardless of storage conditions for up to 60 days, after which temperature and humidity became significant factors affecting survival. In contrast, *Ae. albopictus* egg survival was highly dependent on temperature and humidity, even within the first 30 days of storage [10]. Furthermore, a study in Australia showed that warm and dry conditions are more detrimental to the survival and viability of *Ae. albopictus* eggs [14]. On the other hand, a recent study that used mosquitoes from China found that *Ae. albopictus* eggs were not affected by RH [15].

Intraspecific variations in desiccation resistance have also been observed in *Ae. aegypti*. For instance, eggs collected from two different regions in Tanzania (Buguruni and Msasani) showed significant differences in survival rates when stored under identical laboratory conditions (25 °C and 80% RH). The survival rate of *Ae. aegypti* eggs from Buguruni was up to 28% higher than those from Msasani [12]. These variations suggest that desiccation resistance is a heritable trait, likely influenced by local environmental pressures.

Research on egg survival in unfavorable environmental conditions is crucial for implementing control measures in urban areas. This research helps identify the most susceptible periods for mosquito populations, enabling targeted control interventions. Particularly in Argentina, studies on egg mortality were carried out in the subtropical and temperate regions of the country, but only for *Ae. aegypti* [16,17,18]. However, there is only one work analyzing *Ae. albopictus* eggs since its detection in 1998 [19]. Therefore, this research will allow us to expand knowledge about the biology of these vector species that live in sympatry in Misiones, such as egg survival, and data that will contribute to developing recommendations for prevention and control programs for arbovirus transmission. Due to all this, it is important to compare the desiccation tolerances of *Ae. albopictus* and *Ae. aegypti* in different regions where the species coexist and determine if differences in tolerance to desiccation can explain the local coexistence pattern observed in Misiones. Our objective is to know the survival of *Ae. aegypti* and *Ae. albopictus* eggs under different relative humidity conditions in the laboratory.

## 2. Materials and Methods

### 2.1. Study Area

This study was conducted in Puerto Iguazú city (25°35′52” S, 54°34′55” O), located in Misiones province, a subtropical province of NEA. In this city are the Iguazú National Park and the Iguazú Falls. Puerto Iguazú is located on the triple border between Argentina, Brazil (Iguazú Falls) to the north, and Paraguay to the east (Ciudad del Este); the three countries are naturally divided by the Paraná and Iguazú rivers. The city displays an environmental gradient from urban to rural landscape with remaining patches of natural forest. Due to its proximity to the Iguazú Falls, the city is one of the most important tourist zones in Argentina; it is visited by more than one million people per year and has 99,013 inhabitants in an area of 759 km^2^, being the third largest city in the province [20]. The area belongs to the Paraná jungle ecoregion. The climate is humid subtropical with hot summers and no dry seasons [21]. The coldest months are June and July, with an average minimum temperature of 12 °C and 11 °C, respectively, while the warmest month is January, with an average maximum temperature of 32 °C. Precipitation is abundant throughout the year, with average annual values of 1960 mm. July and August are the driest months, with accumulated precipitation of 107.0 and 83.7 mm, respectively. While in the wettest months (October and December), the accumulated precipitations are 247 and 219 mm, respectively. The average relative humidity is similar throughout the year, with 75.8–88.6% values. The average annual minimum recorded value is approximately 25%, and the maximum is approximately 96.8%. The months of August to November record the lowest minimum humidity values of approximately 18–20% (average climatological values 1991–2020; National Meteorological Service; https://www.smn.gob.ar/, accessed on 1 March 2025).

### 2.2. Eggs Collection

Two populations of mosquitoes were used (*Ae. aegypti* and *Ae. albopictus*), both collected in the larval stage in different dwellings in Puerto Iguazú, seeking to cover different parts of the city. The study city was chosen because it is one of the few locations where *Ae. albopictus* were established and coexisted with *Ae. aegypti* [22]. The colonies and the experiments were established in the Instituto Nacional de Medicina Tropical (INMeT) insectary. After the emergence of the larvae, adults were classified by species and placed in rearing cages. Adults were fed with a 10% sucrose solution, and a blood source (*Gallus* spp.) was offered to females twice a week. The laboratory conditions were maintained at 25 ± 2 °C, with 70 ± 5% RH and a photoperiod of 12/12 (light/dark). After the females were blood-fed, oviposition substrates (paper paddles) were placed inside plastic containers with dechlorinated water, and inside the rearing cages. Eggs were collected weekly on filter papers placed inside plastic containers with dechlorinated water. Filter papers with eggs (F2 generation) were removed from the cages and kept in wet conditions until the beginning of the experiments. Eggs older than 1 month were not used. Two age classes were distinguished: eggs collected during weeks 1–2 (early-laying eggs) and eggs collected during weeks 3–4 (late-laying eggs) [16].

### 2.3. Relative Humidity Experimental Design

Before starting the experiments, the paddles were inspected under a stereomicroscope to count intact eggs and remove any hatched or collapsed eggs. All paddles with at least 10 eggs (mean 31, range 11–94) were used in the experiment, and paddles collected each week were uniformly assigned to treatments. The batches of each treatment comprised several paddles of eggs.

Batches of eggs of *Ae. aegypti* (approximate batch size = 500) and eggs of *Ae. albopictus* (approximate batch size = 200) were exposed to three different RH conditions (35%, 68%, and 82%, respectively). The difference in the number of eggs was because female *Ae. albopictus* laid fewer eggs compared to *Ae. aegypti*. The three RHs were established in different glass desiccators using saturated salt solutions of MgCl_2_-6H_2_O, MgCO_3_, and KCl, respectively [23]. The saturation of the solutions was monitored weekly in each desiccator using a thermo-hygrometer (humidity accuracy: ±5%), and salts were added as necessary. These RH levels were chosen to reflect the natural range of humidity conditions in Puerto Iguazú. Minimum RH values as low as 18–25% are recorded during the dry season (August–November), while typical daily means range from 70 to 90% year-round (average climatological values 1991–2020; National Meteorological Service; https://www.smn.gob.ar/, accessed on 1 March 2025). Thus, 35% RH represents extreme dry conditions, 68% RH simulates intermediate or semi-sheltered urban environments, and 82% RH corresponds to humid, shaded habitats.

Two experiments were carried out. First, we analyzed the survival of egg batches after 1 month in the different RHs for both species. This experiment was repeated three times. After a month, due to the availability of eggs and after analyzing the low mortality of *Ae. aegypti*, another experiment was carried out in which we also analyzed the survival of the eggs after approximately 2 and 3 months.

**Figure 1:**
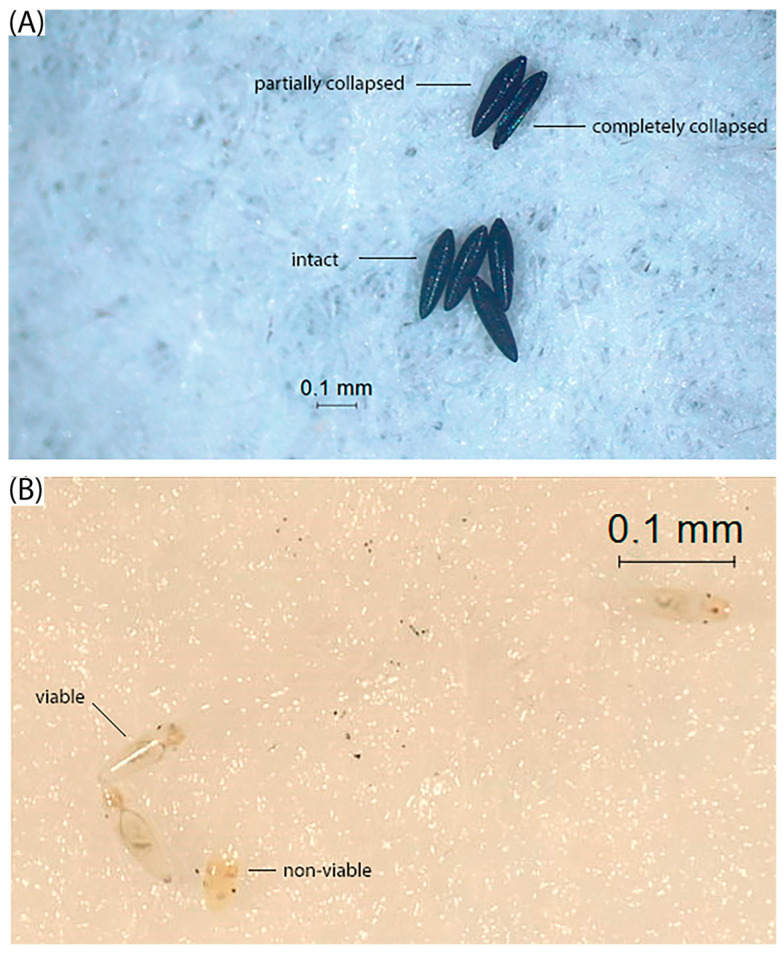
Representative images of (**A**) intact egg, partially collapsed egg, and completely collapsed egg, and (**B**) viable embryo after bleaching (note the eye spots and segmented abdomen), and non-viable embryo showing brown discoloration and lack of clear segmentation.

After 1, 2 or 3 months, as appropriate for each experiment, the remaining eggs in each paddle were counted, and the number of completely collapsed, partially collapsed, or intact eggs was recorded (Figure 1A). All completely collapsed eggs were removed from the paddle and registered as dead. Eggs were transferred to plastic cups containing 150 mL of dechlorinated water to stimulate hatching. Each paddle was immersed separately, and after 48 h, the number of emerged larvae was counted. The paddles were removed from the water, and the immersion was repeated every 4 days. Four immersions were carried out, after which the number of remaining unhatched eggs was negligible. Unhatched eggs remaining on the paddles were bleached with a commercial 50% sodium hypochlorite solution to allow direct observation of the embryos. Embryos that were creamy white with eyespots, a hatched spine, and distinct abdominal segmentation were considered viable. Embryos (Figure 1B) that were yellowish brown or reddish brown were considered nonviable (Figure 1B) [24,25]. In this work, emerged larvae and unhatched viable eggs after successive immersions were considered alive for the corresponding analyses.

### 2.4. Data Analysis

The survival rate of each batch was estimated as the proportion of viable eggs (emerged larvae and unhatched viable eggs) in relation to the total number of eggs present in each paddle (completely collapsed, partially collapsed and intact eggs). To analyze the data collected during the 1-month exposure experiment, a Generalized Linear Model (GLM) was used with a quasibinomial family and logarithmic link function [26], considering the survival of the eggs as the response variable. The independent variables included in the model were Species and RH, as well as their interaction, to evaluate possible combined effects. To identify the significative differences between groups, post hoc comparisons were performed using a Fisher’s LSD rank test with a significance level of *p* < 0.05 [27]. The results were expressed in proportions by the inverse transformation of the link function, thus facilitating the biological interpretation of the observed effects.

Additionally, for the exposure time analysis (1, 2, and 3 months in the desiccator), GLMs were fitted separately for each species, including Exposure Time and HR as independent variables, as well as their interaction. To ensure a balanced design, paddles from treatments with excess replicates were randomly selected to equalize the number of observations in each time category (so that 1-month data have the same number as 2- and 3-month data).

To perform statistical analyses, the freely available software R 4.2.3 (https://www.r-project.org/, accessed on 1 March 2025) and the packages agricolae (LSD. test function), car, multcomp (glht function), and emmeans (emmeans function) were used.

## 3. Results

After all the experiments, the mean percentages of *Ae. aegypti* dead eggs (completely collapsed) were 6.01% at 35%, 2.84% at 68%, and 1.58% at 82%. For *Ae. albopictus*, the percentages were 26.4%, 9.94%, and 6.64%, respectively.

### 3.1. Egg Survival Under Various RH Conditions in 1 Month of Exposure

The mean egg survival after 1 month of exposure showed differences between species: in *Ae. aegypti* it was 92.6%, while in *Ae. albopictus* it reached 66.9%. The GLM model with a quasibinomial family showed that egg survival varies significantly between species, being higher in *Ae. aegypti* than in *Ae. albopictus* (estimator = −3.074, t-value = −6.440, *p* < 0.001). Likewise, RH significantly affects survival in both species (estimator = 0.027, t-value = −3.815, *p* < 0.001), although this effect is less pronounced in *Ae. albopictus* due to the species*RH interaction (estimator = 0.020, t-value = 2.445, *p* = 0.017). At a low RH of 35%, *Ae. aegypti* and *Ae. albopictus* survived at very different rates of approximately 88.0% and 37.6%, respectively. At a mean RH of 68%, the difference between species was smaller, with a survival rate of approximately 95.2% for *Ae. aegypti* and 79.1% for *Ae. albopictus*. At a high RH of 82%, survival was higher in both cases, with a rate of approximately 97.3% for *Ae. aegypti* and 84.1% for *Ae. albopictus* (Table 1).

**Figure 2:**
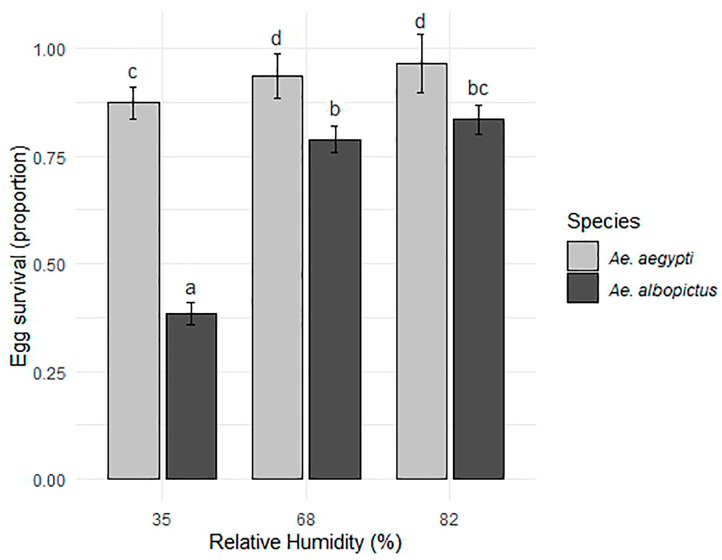
Mean and standard deviation of egg survival (proportion) of *Ae. aegypti* and *Ae. albopictus* as a function of different relative humidity conditions. Different letters above bars indicate statistically significant differences among all species × RH treatment combinations based on Fisher’s test (*p* < 0.05), ordered from lowest to highest survival.

The post hoc test results reveal significant differences between treatments, indicated by the letters assigned to each bar (Figure 2). At low RH levels (35%), *Ae. aegypti* showed significantly higher survival than *Ae. albopictus*. As RH increases (68% and 82%), both species show higher survival rates than the lower RH. Each species’s survival did not vary between 68% and 82% of RH. At 68% RH, *Ae. aegypti* exhibited numerically higher survival than *Ae. albopictus*; this difference was statistically significant, as indicated by letters (“d” and “b”) in the post hoc test. Similarly, significant differences between species were detected at the highest RH level (82%).

### 3.2. Egg Survival Under Various RH Conditions During 1, 2, and 3 Months of Exposure

Overall, *Ae. aegypti* showed higher egg survival under all conditions, with values ranging from 31.0% to 95.7% (Table 2), while *Ae. albopictus* had lower values, between 15.9% and 81.9% (Table 3). In both species, survival decreased with exposure time, especially at 35% RH, with the lowest values recorded at 3 months (*Ae. aegypti*: 31%; *Ae. albopictus*: 16.7%). However, *Ae. aegypti* showed greater resistance to desiccation, maintaining higher survival rates even under adverse conditions. In both cases, 82% RH favored egg survival, but the effect was more pronounced in *Ae. aegypti*.

The results of the GLMs for *Ae. aegypti* showed that RH, exposure time, and the interaction between both variables significantly influence egg survival. RH was found to have a statistically significant effect (estimator = 1.315, t-value = 2.666, *p* < 0.001), indicating that lower humidity levels reduce survival. Furthermore, exposure time (estimator = −2.116, t-value = −5.003, *p* < 0.001) and the interaction between month and RH are significant (estimator = 0.019, t-value = 2.682, *p* = 0.009), suggesting that the impact of RH varies depending on the period evaluated.

**Figure 3:**
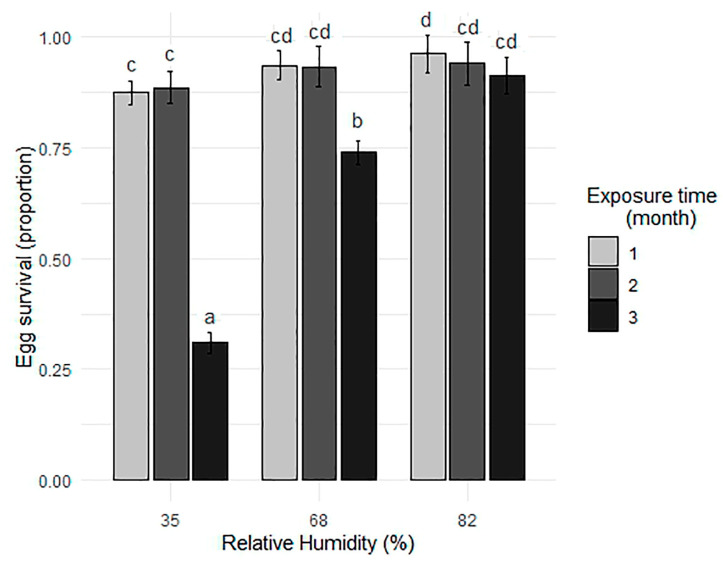
Mean and standard deviation of egg survival (proportion) of *Ae. aegypti* as a function of different relative humidity conditions and exposure times. Different letters above bars indicate statistically significant differences between all exposure time × RH treatment combinations based on Fisher’s test (*p* < 0.05), ordered from lowest to highest survival.

The results of the Fisher’s test indicated significant differences between treatments (Figure 3). At RH of 35%, egg survival of *Ae. aegypti* decreases significantly with exposure time, highest after 1 (letter “a”) and 2 months and lowest after 3 months. At RH of 68% and 82%, survival proportions are higher and show less variation between exposure times. After 3 months of exposure, the survival rate of 68% was the second lowest for this species (letter “b”). In contrast, after 1 month of exposure, the survival rate of 82% was the highest (letter “d”).

Table 2 summarizes the data from the experiment performed for the species. Average survival was higher at the highest RH levels (68% and 82%), with consistently high values at both exposure times. In contrast, the lowest RH (35%) showed lower average survival, especially at the longest exposure time (2 months). Survival was less than 40% after 3 months of exposure to RH of 35%. In contrast, for *Ae. albopictus* egg survival was higher at 1 month of exposure than at 2 and 3 months, with high survival proportions (>60%) only at 68% and 82% RH after 1 month of exposure (Table 3).

The results of the GLM applied to *Ae. albopictus* showed that both RH, exposure time and their interaction significantly affected egg survival. RH had a positive and significant effect (estimator = 0.045, t-value = 4.904, *p* < 0.001), indicating that higher RH levels increase the probability of survival. Exposure time showed a significant negative effect, especially after 2 months (estimator = −1.190, t-value = −4.521, *p* < 0.001) and 3 months (estimator = −1.127, t-value = −4.353, *p* < 0.001), where the decrease in survival was more pronounced. Furthermore, the interaction between RH and exposure time was significant, suggesting that the magnitude of the effect of exposure time depends on the humidity level (estimator = −1.020, t-value = −2.867, *p* = 0.006).

**Figure 4:**
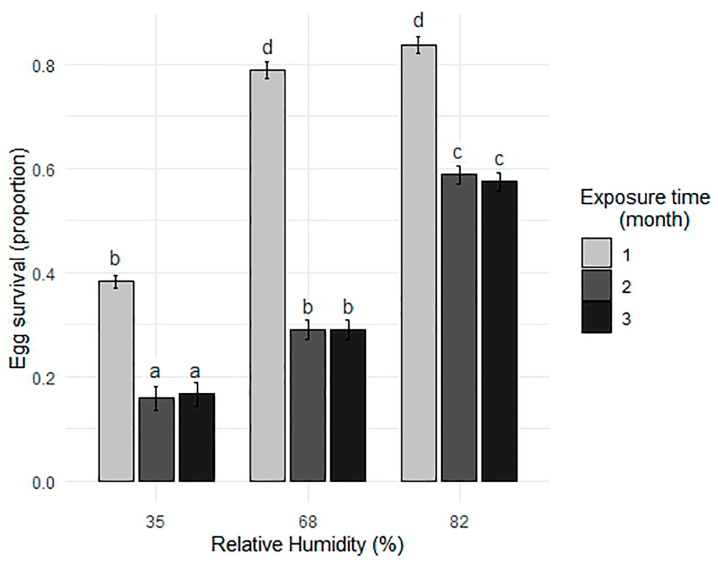
Mean and standard deviation of *Ae. albopictus* egg survival (proportion) as a function of different relative humidity conditions and exposure times. Different letters above bars indicate statistically significant differences between all exposure time × RH treatment combinations based on Fisher’s test (*p* < 0.05), ordered from lowest to highest survival.

A Fisher’s test identified significant differences between treatments for the species. At an RH of 35%, egg survival decreases considerably with exposure time, being higher at 1 month and significantly lower at 2 and 3 months. At an RH of 68%, survival is higher at 1 month and significantly decreases after 2 and 3 months. At the highest RH level (82%), survival is highest at 1 month and remains constant after 2 and 3 months (Figure 4).

## 4. Discussion

This study provides valuable insights into the desiccation tolerance of *Ae. aegypti* and *Ae. albopictus* eggs, two important mosquito vectors of arboviruses, using populations from a subtropical region of Northeastern Argentina, for the first time. Our findings reveal a significant difference in egg survival between the two species under varying RH conditions, with *Ae. aegypti* demonstrating superior resistance to desiccation. This disparity in desiccation resistance could play a crucial role in shaping the distribution and abundance of these species, particularly in the context of climate change.

The observed higher desiccation tolerance of *Ae. aegypti* eggs aligns with previous studies conducted in other regions, suggesting a consistent trait across different populations. It shows significantly higher survival at all RH levels and in all exposure times compared to *Ae. albopictus*, with only a survival rate of less than 37% after 3 months of exposure to 35% RH. Field studies indicate that the eggs of *Ae. aegypti* are resistant to desiccation, surviving for months in a dormant state and hatching when submerged in water [28]. This desiccation tolerance is consistent with that previously reported for *Ae. aegypti* eggs of US origin [10], where low mortality rates were found after 1, 2, and 3 months of exposure to different RH levels. However, a more recent study of mosquitoes from Puerto Rico [29] found that egg-hatching rates were approximately 50% at 25 and 55% RH and 60% at 80% RH, lower than those observed in this study. Resistance can present intraspecific variations [30], so analyzing the species’s behavior in different areas worldwide is crucial.

In addition, our findings align with molecular and developmental evidence demonstrating desiccation resistance in *Ae. aegypti* eggs is closely associated with the formation of the serosal cuticle and chitin biosynthesis during embryogenesis [31,32]. These structural adaptations, specifically the serosa deposition of a chitin-containing layer, are critical for limiting water loss under low humidity conditions. This desiccation tolerance could explain the successful invasion of *Ae. aegypti* into temperate areas worldwide, allowing the species to survive unfavorable periods and establish new populations.

In contrast, the lower desiccation tolerance of *Ae. albopictus* eggs, particularly evident at low RH levels, could limit their expansion and abundance, especially in regions experiencing prolonged droughts. *Aedes albopictus* eggs presented very low survival rates (37.6%) under 35% RH conditions after 1 month of exposure, and their rates decreased as the exposure time increased. Likewise, these results are similar to those found by Asian, Australian, and US populations, who found the eggs of *Ae. albopictus* were much more sensitive to desiccation than those of *Ae. aegypti*, and the latter presented lower mortality rates than *Ae. albopictus* under laboratory conditions [8,10,13] and in the field [33]. However, a recent study of US populations [29] found similar hatching rates for both *Ae.* species (*aegypti* and *albopictus*), the second species presenting higher values than those observed previously and in this study. At 25% and 55% RH, the hatching rates were approximately 50%. In addition, they found that *Ae. albopictus* had the highest hatching rate at 80% RH. Previous studies have observed that egg mortality in this species is lowest in wetter environments [10,34].

In turn, ref. [14] demonstrated in Australia that hot and dry conditions are more detrimental to the egg viability of *Ae. albopictus*. This study indicates that egg survival of this species depends on the level of dryness but, at the same time, on their duration [10]. That is, dry times of the year or even prolonged periods of drought could affect the abundance of these populations, especially that of *Ae. albopictus*. In addition, this could indicate that when the eggs hatch after a period without rain, there should be a reduction in the abundance of *Ae. albopictus* larvae compared to *Ae. aegypti*. This aligns with the findings of [19], who observed dormancy in *Ae. albopictus* populations from northeastern Argentina is less pronounced than temperate populations; our study further supports their results. This lower intensity of dormancy, as evidenced by their reduced desiccation tolerance, suggests that *Ae. albopictus* may struggle to expand its range into more temperate regions, particularly under climatic stress conditions such as low humidity [35].

Ref. [36] conducted an interspecific competition experiment in *Ae. aegypti* and *Ae. albopictus* in the adult, larva, and egg stages during drying treatments and fluctuating amounts of water. They found that interspecific competition was highly asymmetric. In the more humid environment, interspecific competition had a significant negative effect on *Ae. aegypti*, but in the drier environment, interspecific competition had a large negative effect on *Ae. albopictus*, and a relatively small impact on *Ae. aegypti*. The leading cause of the change in competitive advantage appeared to be a further increase in egg mortality of *Ae. albopictus* under dry conditions, compared to *Ae. aegypti*. This could indicate that the resistance of the eggs to desiccation could be one of the factors that facilitate the coexistence of these species. At the regional or local level, there are likely to be microhabitats that differentially favor the presence of one species or another, favoring habitat segregation [37,38].

Climatic conditions and their effects on egg survival could explain much of the variation observed in the current distribution of these two species in Argentina. A key question for the species is whether environmental factors like temperature, relative humidity, and photoperiod account for its limited distribution in Argentina. The only laboratory study was developed in Puerto Iguazú, which evaluated the effects of different temperature conditions on dormancy induction [19]. They discussed the fact that temperature appears not to be the limiting factor in the geographic expansion of the species into temperate regions. However, RH seems to be a possible factor limiting this mosquito distribution since, according to our study, low RH significantly affects egg survival. South American populations of this mosquito originating from tropical Asian populations do not exhibit diapause [39]. Ref. [19] found that the local population of Puerto Iguazú is capable of entering diapause and hypothesized that a tropical origin like the Brazilian populations [40] could be for the Argentine populations, but more studies should be carried out. According to [41], RH is closely related to the induction of diapause or quiescence and the consequent egg hatching. In turn, the low resistance to egg desiccation that was observed in *Ae. albopictus* could not only limit its expansion but also prevent its passive transport through the country, explaining its limited distribution more than 20 years after its introduction in this country [22]. In addition, mosquitoes with desiccation-resistant eggs would be more likely to establish themselves in non-native habitats compared to mosquito species with desiccation-susceptible eggs, presumably because desiccation-resistant eggs are more likely to survive long-term and long-distance transport [9].

These habitat selection patterns are influenced by abiotic factors, such as water availability and sun exposure, and sociocultural practices that modulate the generation and maintenance of breeding sites. Storing water in open containers or managing solid waste, among others, can create environments conducive to the proliferation of both species [42], but in different proportions depending on local conditions. Tires exposed to sunlight, which usually accumulate small amounts of water, represent a favorable habitat for *Ae. aegypti* due to their ability to deposit eggs with remarkable resistance to desiccation, allowing them to survive in conditions of greater exposure and water fluctuation. In contrast, gardens with shaded and well-irrigated pots, and ponds or pools with a relatively larger water surface generate optimal conditions for the oviposition and development of *Ae. albopictus*, whose preference for humid and shaded environments is associated with areas with greater vegetation cover [43,44]. These dynamics highlight the importance of integrating environmental factors and human habits when designing vector control strategies aimed at minimizing arbovirus transmission.

Our findings have significant implications for understanding the impact of climate change on mosquito populations. As global temperatures rise, many regions, including Puerto Iguazú, are expected to experience more frequent and severe droughts. The lower desiccation resistance of *Ae. albopictus* suggests these changing conditions might negatively impact this species, potentially limiting its distribution and abundance. Conversely, *Ae. aegypti*, being more resilient, might become the dominant species in drier regions. This shift in species dominance could have implications for the transmission dynamics of arboviruses, as different mosquito species exhibit varying vector competences for different pathogens. Future studies should explore the potential impact of climate change on the competitive balance between *Ae. aegypti* and *Ae. albopictus*, their future distributions, and their implications for arbovirus transmission.

In conclusion, this study highlights the importance of RH as a key factor influencing the life cycle of *Ae. aegypti* and *Ae. albopictus*. Greater variation in this climate can help explain survival dynamics and population persistence in their native and invaded habitats. While the role of RH in mosquito biology is well established, the novelty of our work lies in its regionally focused, comparative approach using sympatric populations from northeastern Argentina. By characterizing interspecific differences in desiccation tolerance under controlled RH conditions, we provide mechanistic insight into species distribution and microhabitat preferences in a subtropical context.

Our findings suggest that differential desiccation tolerance may also contribute to habitat partitioning between these species, as previously observed in field studies (e.g., [45,46]). This has important operational implications, as it indicates that environmental conditions may drive species-specific habitat selection—information that can be directly applied to the design of more spatially and temporally targeted vector control strategies. Furthermore, these results are relevant for anticipating potential shifts in vector composition and arbovirus transmission under future climate change scenarios. This research thus contributes both to fundamental mosquito ecology and to the development of more effective, evidence-based vector control interventions.

## Figures and Tables

**Table 1:** Summary of data with count (N), mean egg survival, standard deviation (SD) and 95% confidence interval (CI). N corresponds to the number of paddles placed in each treatment.

Species	Relative Humidity (%)	N	Survival	SD	CI
*Aedes aegypti*	35	11	0.880	0.017	0.039
	68	11	0.952	0.016	0.036
	82	11	0.973	0.007	0.016
*Aedes albopictus*	35	11	0.376	0.045	0.101
	68	11	0.791	0.016	0.036
	82	11	0.841	0.024	0.053

**Table 2:** Summary of data for *Ae. aegypti* with count (N), mean egg survival, standard deviation (SD) and 95% confidence interval (CI). N corresponds to the number of paddles placed in each treatment.

Exposure Time (Month)	Relative Humidity (%)	N	Survival	SD	CI
1	35	6	0.879	0.019	0.050
1	68	6	0.953	0.014	0.037
1	82	6	0.957	0.011	0.028
2	35	6	0.886	0.042	0.109
2	68	6	0.933	0.020	0.051
2	82	6	0.941	0.023	0.058
3	35	6	0.310	0.074	0.189
3	68	6	0.739	0.105	0.267
3	82	6	0.913	0.029	0.074

**Table 3:** Summary of data for *Ae. albopictus* with count (N), mean egg survival, standard deviation (SD) and 95% confidence interval (CI). N corresponds to the number of paddles placed in each treatment.

Exposure Time (Month)	Relative Humidity (%)	N	Survival	SD	CI
1	35	6	0.397	0.061	0.156
1	68	6	0.790	0.018	0.047
1	82	6	0.819	0.040	0.102
2	35	6	0.159	0.007	0.017
2	68	6	0.290	0.018	0.046
2	82	6	0.588	0.034	0.089
3	35	6	0.167	0.026	0.067
3	68	6	0.290	0.045	0.117
3	82	6	0.574	0.053	0.136

## Data Availability

The data sets generated and/or analyzed during the current study are not publicly available due to belonging to the MEM doctoral thesis, but are available from the corresponding author upon reasonable request.
